# AI-Driven Innovations for Early Sepsis Detection by Combining Predictive Accuracy With Blood Count Analysis in an Emergency Setting: Retrospective Study

**DOI:** 10.2196/56155

**Published:** 2025-01-24

**Authors:** Tai-Han Lin, Hsing-Yi Chung, Ming-Jr Jian, Chih-Kai Chang, Hung-Hsin Lin, Chiung-Tzu Yen, Sheng-Hui Tang, Pin-Ching Pan, Cherng-Lih Perng, Feng-Yee Chang, Chien-Wen Chen, Hung-Sheng Shang

**Affiliations:** 1 Division of Clinical Pathology, Department of Pathology Tri-Service General Hospital, National Defense Medical Center Taipei Taiwan; 2 Division of Infectious Diseases and Tropical Medicine, Department of Internal Medicine Tri-Service General Hospital, National Defense Medical Center Taipei Taiwan; 3 Division of Pulmonary and Critical Care Medicine, Department of Internal Medicine Tri-Service General Hospital, National Defense Medical Center Taipei Taiwan

**Keywords:** sepsis, artificial intelligence, critical care, complete blood count analysis, CBC analysis, artificial intelligence clinical decision support systems, AI-CDSS

## Abstract

**Background:**

Sepsis, a critical global health challenge, accounted for approximately 20% of worldwide deaths in 2017. Although the Sequential Organ Failure Assessment (SOFA) score standardizes the diagnosis of organ dysfunction, early sepsis detection remains challenging due to its insidious symptoms. Current diagnostic methods, including clinical assessments and laboratory tests, frequently lack the speed and specificity needed for timely intervention, particularly in vulnerable populations such as older adults, intensive care unit (ICU) patients, and those with compromised immune systems. While bacterial cultures remain vital, their time-consuming nature and susceptibility to false negatives limit their effectiveness. Even promising existing machine learning approaches are restricted by reliance on complex clinical factors that could delay results, underscoring the need for faster, simpler, and more reliable diagnostic strategies.

**Objective:**

This study introduces innovative machine learning models using complete blood count with differential (CBC+DIFF) data—a routine, minimally invasive test that assesses immune response through blood cell measurements, critical for sepsis identification. The primary objective was to implement this model within an artificial intelligence–clinical decision support system (AI-CDSS) to enhance early sepsis detection and management in critical care settings.

**Methods:**

This retrospective study at Tri-Service General Hospital (September to December 2023) analyzed 746 ICU patients with suspected pneumonia-induced sepsis (supported by radiographic evidence and a SOFA score increase of ≥2 points), alongside 746 stable outpatients as controls. Sepsis infection sources were confirmed through positive sputum, blood cultures, or FilmArray results. The dataset incorporated both basic hematological factors and advanced neutrophil characteristics (side scatter light intensity, cytoplasmic complexity, and neutrophil-to-lymphocyte ratio), with data from September to November used for training and data from December used for validation. Machine learning models, including light gradient boosting machine (LGBM), random forest classifier, and gradient boosting classifier, were developed using CBC+DIFF data and were assessed using metrics such as area under the curve, sensitivity, and specificity. The best-performing model was integrated into the AI-CDSS, with its implementation supported through workshops and training sessions.

**Results:**

Pathogen identification in ICU patients found 243 FilmArray-positive, 411 culture-positive, and 92 undetected cases, yielding a final dataset of 654 (43.8%) sepsis cases out of 1492 total cases. The machine learning models demonstrated high predictive accuracy, with LGBM achieving the highest area under the curve (0.90), followed by the random forest classifier (0.89) and gradient boosting classifier (0.88). The best-performing LGBM model was selected and integrated as the core of our AI-CDSS, which was built on a web interface to facilitate rapid sepsis risk assessment using CBC+DIFF data.

**Conclusions:**

This study demonstrates that by providing streamlined predictions using CBC+DIFF data without requiring extensive clinical parameters, the AI-CDSS can be seamlessly integrated into clinical workflows, enhancing rapid, accurate identification of sepsis and improving patient care and treatment timeliness.

## Introduction

The World Health Organization classifies sepsis as a critical global health issue, responsible for approximately 20% of all deaths worldwide in 2017, with 48.9 million cases and 11 million fatalities [1,2]. Sepsis, a life-threatening condition triggered by the body’s response to infection, leads to severe consequences, such as septic shock, organ failure, and even death if not promptly managed [3]. The mortality rate for sepsis is alarmingly high at 32.2%, and it rises significantly to 38.5% for those who develop septic shock, as reported in a meta-analysis [4]. It predominantly affects high-risk groups such as older adults, intensive care unit (ICU) patients, and those with chronic conditions or compromised immune systems [5]. The prevalence of sepsis, which is intensified by drug-resistant infections, highlights the need for more effective prevention and treatment methods [6] and rapid diagnostic strategies [7], particularly in optimizing their implementation in clinical settings [8]. The key to mitigating the heavy health toll of sepsis lies in its efficient management and, crucially, early detection, which significantly enhances the probability of recovery and diminishes its overall detrimental health impact [1,9].

According to the Surviving Sepsis Campaign, sepsis diagnosis relies on the Sequential Organ Failure Assessment (SOFA) score, which evaluates organ dysfunction resulting from infection by assessing 6 components: respiratory function through the ratio of partial pressure of oxygen in arterial blood to the fraction of inspiratory oxygen concentration, coagulation through the platelet count, liver function with bilirubin levels, cardiovascular status through hypotension or the use of vasopressors, central nervous system function using the Glasgow Coma Scale, and renal function measured through creatinine levels or urine output [3]. An increase of 2 or more points in the SOFA score, combined with suspected infection, indicates sepsis [3,10]. For rapid assessments outside of ICUs, the quick SOFA criteria, altered mental state, respiratory rate of ≥22 breaths per minute, or low blood pressure (systolic blood pressure≤100 mm Hg) are used for prompt evaluation, which is critical for reducing sepsis-related health impacts [3,10]. Alongside identifying and addressing the source of infection or removing suspected infected routes or devices, immediate administration of broad-spectrum antimicrobial agents within 1 hour of detecting sepsis is crucial for effective management [3,10,11]. Rapid intravenous crystalloid resuscitation and ongoing fluid adjustments are essential to maintain circulatory volume and ensure organ perfusion [3,10]. Vasopressors, such as norepinephrine, should be applied if a mean arterial pressure of at least 65 mm Hg cannot be maintained [3,10]. Dynamic monitoring of stroke volume, pulse pressure variations, and respiratory status is essential for effective treatment, enabling precise, real-time adjustments to fluid therapy [3,10].

Traditional diagnostic methods such as clinical assessments, laboratory evaluations, and patient histories, while foundational, often lack the immediacy and specificity required to promptly confirm infections [3,5,12]. The progression from infection to sepsis can be insidious, with initial clinical manifestations that are highly variable and influenced by both host and pathogen factors [10]. This variability can be particularly detrimental, as rapid identification within the first crucial hours is vital; delays in diagnosis or the initiation of antibiotic treatment can lead to rapid deterioration in the patient’s condition, significantly worsening the prognosis [10]. While existing machine learning models have sought to reduce these delays by expediting the diagnosis of sepsis, many rely on complex clinical factors that can still delay results or affect accuracy [13-15]. In addition, blood cultures, though critical for identifying pathogens, could take days and are prone to false negatives, further complicating the timely management of sepsis [3,5,12]. This highlights a critical need for simpler, more effective diagnostic approaches that can be swiftly applied and have high reliability.

Addressing these challenges, our research introduces a novel machine learning model centered on complete blood count with differential (CBC+DIFF) data, a routine and minimally invasive test that measures the levels of various blood cell types, including white blood cells, red blood cells, and platelets. This approach was selected for its broad clinical use, rapid analytical time, and ability to evaluate immune response through white blood cell differential analysis (such as neutrophils and lymphocytes), which are crucial for early sepsis detection. This makes it an accessible and cost-effective tool for diagnostics, allowing us to simplify the diagnostic process without sacrificing accuracy. The primary objective of this study was to develop and implement an artificial intelligence–clinical decision support system (AI-CDSS) that uses this model to provide rapid and precise indications of sepsis. This system aims to facilitate earlier intervention and potentially improve patient outcomes across various medical settings, from outpatient departments to emergency departments, with a particular focus on high-stakes environments such as ICUs, where timely decisions are crucial.

## Methods

### Study Focus and Data Collection

In our retrospective study conducted at the ICU of Tri-Service General Hospital from September to December 2023, we focused on cases suspected of pneumonia-induced sepsis, as approved by the hospital’s institutional review board. The study site, a major teaching hospital affiliated with the National Defense Medical Center in Taipei, Taiwan, is a facility with approximately 1800 beds, serving as the primary referral center for 3 branch hospitals in northern Taiwan. This comprehensive medical center provides advanced medical facilities and multidisciplinary care to both civilian and military patients, while its branch hospitals focus on chronic condition management and refer complex acute cases, including sepsis, to the main facility. This centralized health care network structure enabled us to collect data from a diverse patient population across various clinical settings. Our data were exclusively derived from the electronic medical records of patients who were either transferred from the general ward owing to deteriorating conditions or directly admitted from the emergency department owing to severe initial clinical presentations. These records include admission notes, progress notes, laboratory results, and radiographic findings. We included only those patients whose records indicated pneumonia as the suspected cause of sepsis, corroborated by radiographic evidence consistent with pneumonia and an increase in the SOFA score of 2 or more points, indicating significant organ dysfunction. Specifically, we extracted and analyzed data on CBC+DIFF, sputum cultures, blood cultures, and FilmArray analyses. These selected data points formed our core dataset used to analyze the diagnostic and management strategies for pneumonia-induced sepsis in the ICU.

### Control Group Selection

In addition to the groups directly involved in the sepsis investigation, the study included a control group of 746 randomly selected outpatients from the same hospital, enrolled during the same period. These patients typically visited the hospital for routine follow-up appointments. On their appointment days, they first stopped at our blood draw station, where standard blood tests were performed. The results of these tests were available to their physicians within 1 hour, ensuring that the most current health data would be reviewed during their visit. Data from these patients were obtained from the hospital’s electronic medical records. These records confirmed that none of these individuals required subsequent transfer to the emergency department for acute issues, verifying their stable health condition at the time of their visit. CBC+DIFF data were collected specifically for this group.

### Ethical Considerations

Our retrospective study was conducted following strict ethical guidelines approved by the Tri-Service General Hospital’s Institutional Review Board (approval C202305073). The study adhered to the principles outlined in the Declaration of Helsinki and relevant local ethical regulations. The study involved analyzing anonymized patient data to ensure privacy and confidentiality and was exempt from the requirement for patient consent under local and international ethical regulations. No compensation was provided to the patients as no direct interaction was involved.

### Sepsis Identification and Classification Criteria

#### Criteria for Confirming Sepsis

We initially considered all ICU patients with suspected sepsis. Confirmation depended on positive sputum or blood cultures, or FilmArray test results [16], in conjunction with clinical symptoms and identified infection sources.

#### Exclusion Parameters

Cases lacking data or with poor sample quality (including blood samples with volumes less than 10 mL, sputum samples less than 1 mL, or showing contamination indicators such as squamous cell count>10/low power field or mixed bacterial flora), impacting the trustworthiness of culture reports, were excluded.

#### Determination of Nonseptic Status

Patients with negative results in both culture and FilmArray tests were classified as nonseptic.

### Data Processing and Model Formulation

#### Data Organization

We meticulously organized the patient data using Python’s *Pandas* framework (Python Software Foundation) to enhance data handling and analysis efficiency.

#### Selection of Features

Our approach involved a comprehensive analysis of hematological parameters from the CBC+DIFF data using the Sysmex XN-9100 system, focusing on parameters relevant to sepsis pathology. These included white blood cell count (10^3^/µL), red blood cell count (10^6^/µL), hemoglobin (g/dL), hematocrit (%), mean corpuscular volume (fL), mean corpuscular hemoglobin (pg), mean corpuscular hemoglobin concentration (g/dL), platelet count (10^3^/µL), neutrophil percentage (%), lymphocyte percentage (%), monocyte percentage (%), eosinophil percentage (%), basophil percentage (%), immature granulocyte count (10^3^/µL), immature granulocyte percentage (%), neutrophil side scatter light intensity, neutrophil cytoplasmic complexity, and neutrophil-to-lymphocyte ratio. Special attention was paid to neutrophil side scatter light intensity and neutrophil cytoplasmic complexity regarding their insights into neutrophil characteristics. Heat map visualization aided in identifying the most significant features of our predictive model and in discarding less relevant features to optimize the dataset for targeted analysis. In addition, we used Shapley additive explanations (SHAP) values to interpret feature importance and contribution to the model’s output. SHAP summary plots generated with the *TreeExplainer* illustrated the impact of individual features on sepsis risk predictions, enhancing our understanding of the model’s decision-making process and ensuring interpretability and reliability.

### Model Development and Evaluation

#### Data Segmentation

The dataset was divided, with data from September to November forming the training set and data from December used for validation.

#### Using Machine Learning Algorithms

We used a diverse range of machine learning models for the extensive analysis of predictive capabilities. These included logistic regression (LR), linear discriminant analysis, the random forest classifier (RFC), the gradient boosting classifier (GBC), the AdaBoost classifier, extreme gradient boosting (XGBoost), and the light gradient boosting machine (LGBM). Each model was selected to provide a comprehensive perspective on the individual and collective abilities to accurately predict sepsis.

#### Assessment of Predictive Accuracy

These models were stringently tested using receiver operating characteristic curves, the area under the curve (AUC), sensitivity, specificity, positive predictive value (PPV), negative predictive value (NPV), and the *F*_1_-score to measure the accuracy of sepsis prediction. We also evaluated the models using the Brier score and Brier skill score (BSS) to assess the accuracy and calibration of probabilistic predictions. The Brier score measures the mean squared difference between predicted probabilities and actual outcomes, with lower scores indicating better accuracy. The BSS compares the model’s performance against a baseline model using LR, which provides a more representative benchmark. Values above 0 for the BSS indicate superior performance.

#### Clinical Utility Evaluation

To select the best-performing model for accurate sepsis prediction, we validated the models using data collected in December and evaluated key performance metrics, including AUC, sensitivity, specificity, PPV, NPV, and *F*_1_-score. The optimal model was saved using Python’s *joblib* package for seamless implementation. For clinical deployment, we created a web-based, AI-CDSS interface that allows clinicians to initiate sepsis risk assessment by entering a patient ID. The system retrieves laboratory values, sends them to a Python Flask server for analysis, and then displays the calculated sepsis probability. In addition, health care professionals received comprehensive support through group workshops and online training sessions. Feedback collected through surveys and interviews was used to continuously refine the tool, enhancing its usability and integration into clinical workflows.

## Results

### Patient Demographics and Analysis

**Figure 1:**
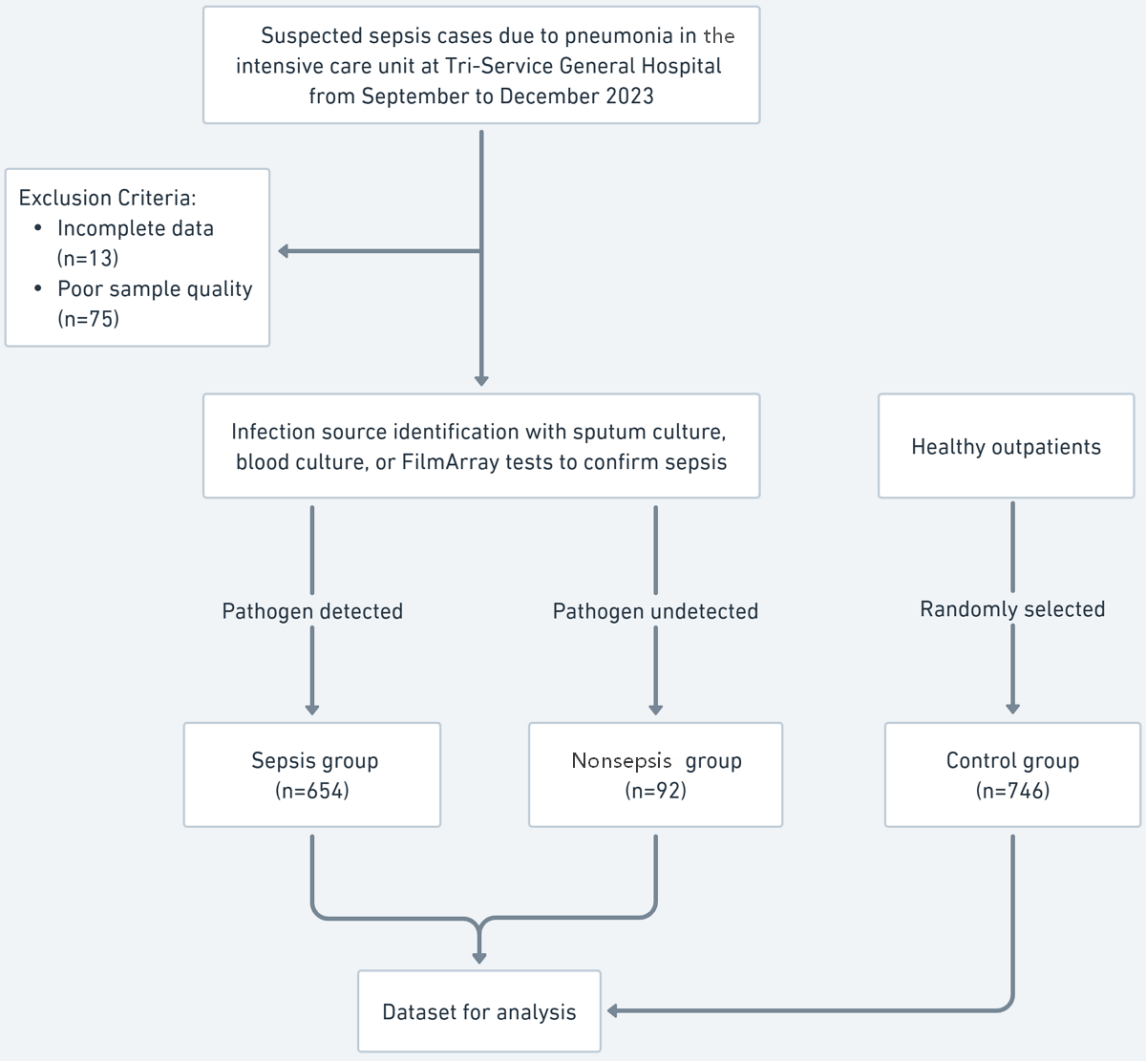
Patient selection flowchart.

Our study included 746 ICU patients and a control group of 746 randomly selected healthy outpatients, which served as a normative benchmark. Among the ICU patients, 654 were classified in the sepsis group based on confirmed pathogen detection: 243 were identified through FilmArray tests and 411 through culture tests. The remaining 92 patients, initially suspected of sepsis, were reclassified into the nonsepsis group due to the absence of pathogen detection, suggesting alternative etiologies for their symptoms. This classification ultimately yielded a final dataset consisting of 654 (43.8%) sepsis cases out of a total of 1492 cases (Figure 1). We compiled the demographic and clinical characteristics, including the CBC+DIFF parameters, for each group, as detailed in Table 1.

**Table 1:** Demographic and hematological characteristics of study groups.

Parameter	Sepsis group (n=654)	Nonsepsis group (n=92)	Control group (n=746)
Age (years), mean (SD)	69.04 (16.4)	67.98 (18.17)	55.52 (17.6)
Sex (male), n/N (%)	425/654 (64.98)	57/92 (61.96)	482/746 (64.61)
WBC^a^ (10^3^/µL), mean (SD)	10.06 (5.84)	11.34 (6.15)	6.88 (3.67)
RBC^b^ (10^6^/µL), mean (SD)	3.51 (0.82)	3.35 (0.71)	4.47 (0.77)
Hb^c^ (g/dL), mean (SD)	10.26 (2.14)	9.89 (2.02)	13.05 (2.14)
HCT^d^ (%), mean (SD)	31.62 (6.45)	30.15 (6.18)	40.01 (6.04)
MCV^e^ (fL), mean (SD)	90.76 (7.35)	90.45 (6.72)	90.21 (8.33)
MCH^f^ (pg), mean (SD)	29.46 (2.7)	29.68 (2.3)	29.42 (3.31)
MCHC^g^ (g/dL), mean (SD)	32.45 (1.45)	32.83 (1.39)	32.56 (1.26)
Platelet count (10^3^/µL), mean (SD)	187.63 (110.81)	196.45 (106.68)	238.41 (87.26)
Neutrophil (%), mean (SD)	78.45 (13.85)	84.62 (9.99)	60.92 (12.82)
Lymphocyte (%), mean (SD)	13.43 (10.9)	9.31 (7.27)	29.13 (11.86)
Monocyte (%), mean (SD)	6.18 (4.43)	5.08 (3.35)	6.57 (2.52)
Eosinophil (%), mean (SD)	1.65 (4.07)	0.76 (1.58)	2.79 (2.7)
Basophil (%), mean (SD)	0.3 (0.26)	0.23 (0.19)	0.6 (0.39)
Immature granulocytes (10^3^/µL), mean (SD)	0.17 (0.34)	0.25 (0.56)	0.06 (0.2)
Immature granulocytes (%), mean (SD)	1.51 (2.41)	1.78 (2.68)	0.64 (1.4)
Neutrophil side scatter light intensity, mean (SD)	52.38 (9.22)	52.7 (5.41)	47.03 (3.34)
Neutrophil cytoplasmic complexity, mean (SD)	688.13 (184.1)	681.86 (110.85)	608.05 (79.31)
Neutrophil to lymphocyte ratio, mean (SD)	13.76 (39.89)	20.81 (51.03)	3.19 (4.35)

^a^WBC: white blood cell count.

^b^RBC: red blood cell count.

^c^Hb: hemoglobin.

^d^HCT: hematocrit.

^e^MCV: mean corpuscular volume.

^f^MCH: mean corpuscular hemoglobin.

^g^MCHC: mean corpuscular hemoglobin concentration.

In Figure 1, a comprehensive flowchart illustrates the patient selection process used in our study. It outlines the initial screening of ICU patients with suspected pneumonia; the criteria used to confirm sepsis (such as positive results from sputum or blood cultures or FilmArray tests); and how patients were categorized into groups of confirmed sepsis, suspected nonsepsis, and outpatient control. In addition, this flowchart delineates the number of patients at each stage and the reasons for exclusion.

### Data Visualization and Feature Analysis

**Figure 2:**
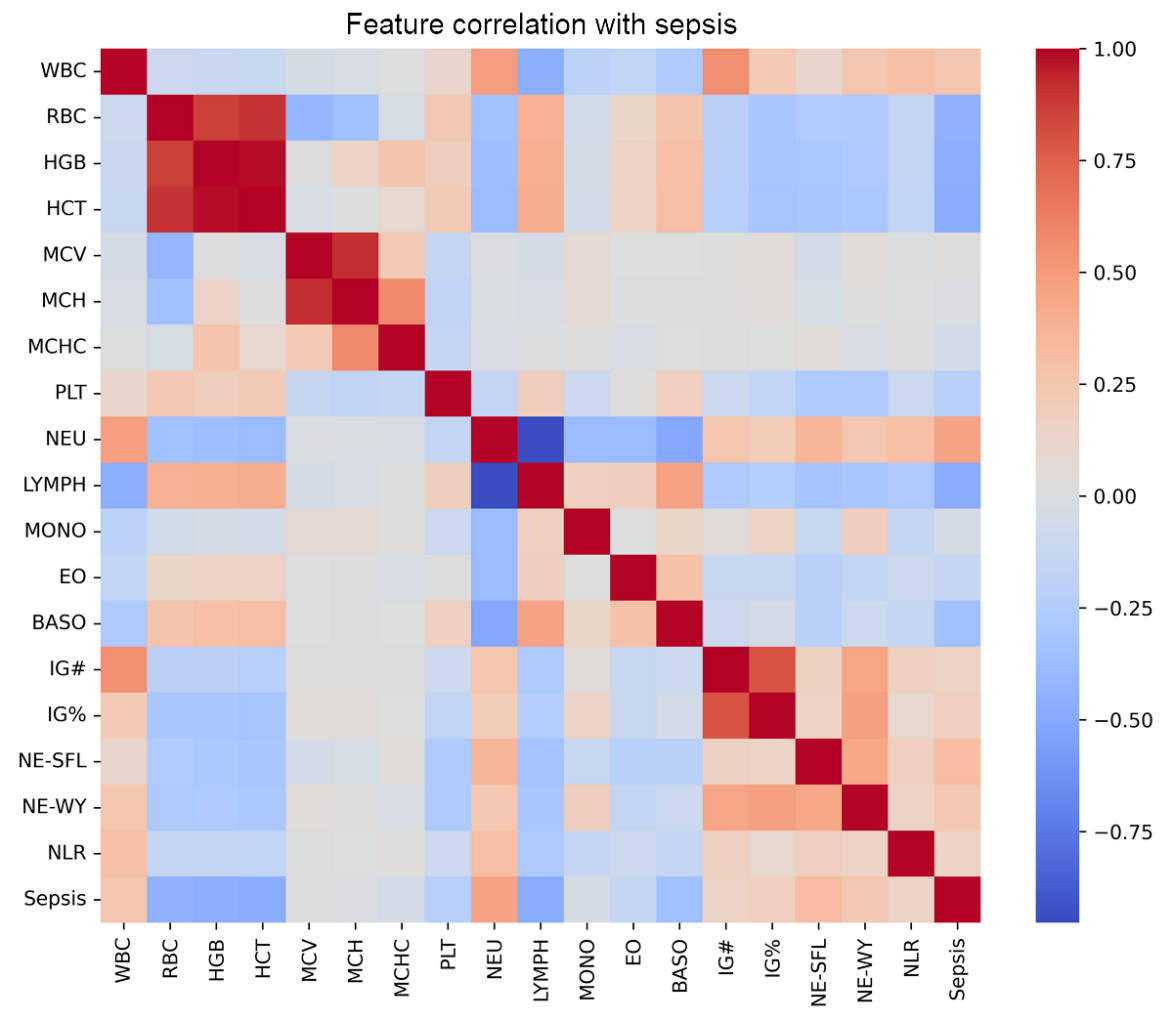
Correlation heat map of complete blood count with differential (CBC+DIFF) parameters and sepsis. BASO: basophil (%); EO: eosinophil (%); HCT: hematocrit (%); HGB: hemoglobin (g/dL); IG#: immature granulocytes (10^3^/µL); IG%: immature granulocytes (%); LYMPH: lymphocyte (%); MCH: mean corpuscular hemoglobin (pg); MCHC: mean corpuscular hemoglobin concentration (g/dL); MCV: mean corpuscular volume (fL); MONO: monocyte (%); NE-SFL: neutrophil side scatter light intensity; NEU: neutrophil (%); NE-WY: neutrophil cytoplasmic complexity; NLR: neutrophil-to-lymphocyte ratio; PLT: platelet count (10^3^/µL); RBC: red blood cell count (10^6^/µL); WBC: white blood cell count (10^3^/µL).

For feature selection, we used heat maps to graphically represent the correlation between various CBC+DIFF parameters and sepsis, as shown in Figure 2. This visualization technique enabled us to eliminate parameters, such as the mean corpuscular hemoglobin concentration, mean corpuscular volume, and monocyte percentage, which had minimal impact on our model’s predictions. The SHAP summary plot, as shown in Figure S1 in Multimedia Appendix 1, further revealed that lymphocyte percentage was the most influential feature, with high values decreasing the sepsis risk. Neutrophil percentage and neutrophil-to-lymphocyte ratio strongly correlated with an increased sepsis risk. Features such as mean corpuscular hemoglobin concentration, mean corpuscular volume, and monocyte percentage also show the least impact, aligned with the interpretation from Figure 2. This confirms the critical role of specific blood parameters in sepsis detection and enhances our model’s interpretability and clinical relevance.

In Figure 2, the heat map depicts the correlations between various CBC+DIFF parameters and the occurrence of sepsis. This effectively shows the relevance of each parameter in predicting sepsis and serves as a crucial tool for identifying and selecting key features for the machine learning model. The color gradients in the heat map correspond to the varying strengths of these correlations.

### Machine Learning Model Performance

**Figure 3:**
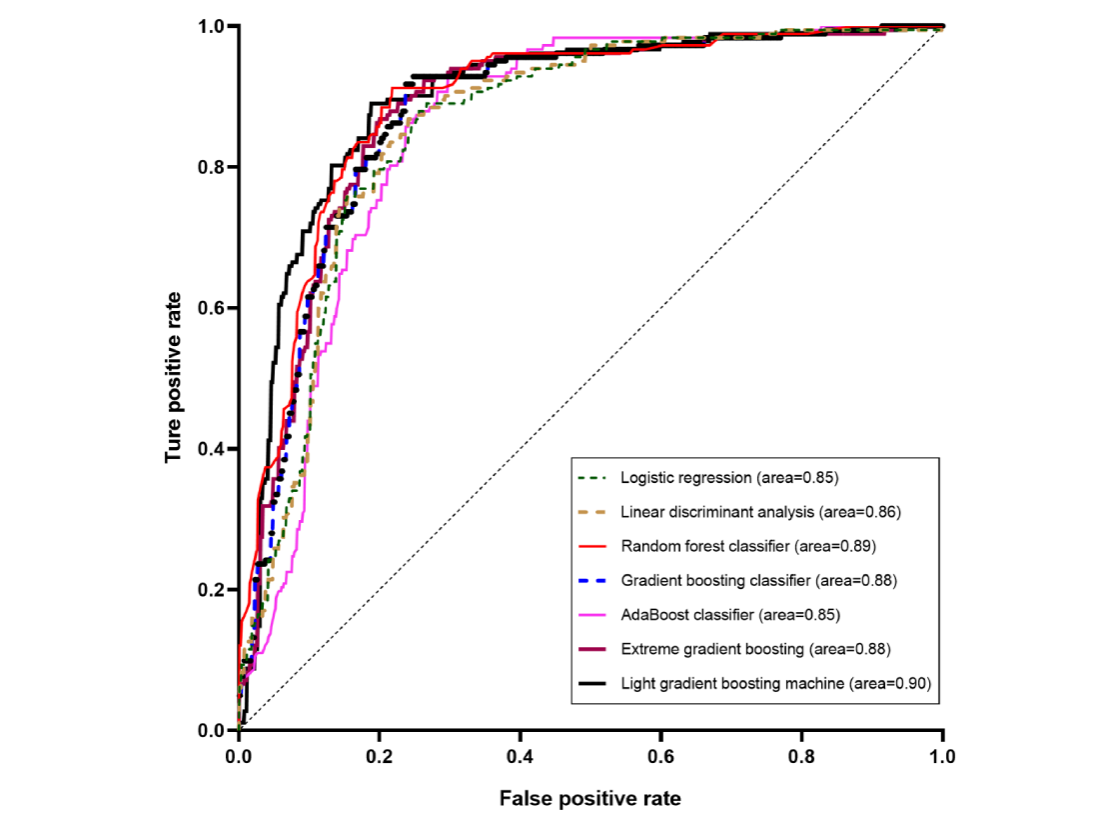
Receiver operating characteristic curves of the evaluated machine learning models.

Our analysis of the machine learning models for detecting sepsis yielded significant results, as detailed in Table 2 and illustrated in Figure 3. The GBC and RFC models exhibited superior predictive performance, with the GBC model achieving a perfect training AUC of 0.99 and a testing AUC of 0.88. The RFC model demonstrated excellent reliability, with a training AUC of 0.94 and a testing AUC of 0.89.

**Table 2:** Overview of machine learning models’ performance.

Model	Training AUC^a^	Testing AUC	Accuracy	Sensitivity	Specificity	PPV^b^	NPV^c^	*F*_1_-score	Brier Score	BSS^d^
LR^e^	0.86	0.85	0.80	0.80	0.80	0.74	0.85	0.77	0.15	Ref
LDA^f^	0.86	0.86	0.80	0.80	0.80	0.73	0.85	0.76	0.15	0.17%
RFC^g^	0.94	0.89	0.81	0.84	0.79	0.73	0.88	0.78	0.13	15.79%
GBC^h^	0.99	0.88	0.83	0.86	0.81	0.75	0.89	0.80	0.13	12.54%
ABC^i^	0.90	0.85	0.79	0.77	0.80	0.72	0.83	0.74	0.22	–47.29%
XGBoost^j^	0.97	0.88	0.80	0.81	0.80	0.73	0.86	0.77	0.13	14.33%
LGBM^k^	0.99	0.90	0.83	0.85	0.82	0.76	0.89	0.80	0.13	10.38%

^a^AUC: area under the curve.

^b^PPV: positive predictive value.

^c^NPV: negative predictive value.

^d^BSS: Brier skill score.

^e^LR: logistic regression.

^f^LDA: linear discriminant analysis.

^g^RFC: random forest classifier.

^h^GBC: gradient boosting classifier.

^i^ABC: AdaBoost classifier.

^j^XGBoost: extreme gradient boosting.

^k^LGBM: light gradient boosting machine.

Moreover, the LGBM model surpassed the others in terms of testing accuracy, scoring a testing AUC of 0.90, highlighting its analytical efficiency. The XGBoost model also showed substantial potential for accurately predicting sepsis with an AUC of 0.88.

The GBC model showed a high PPV of 0.75, indicating its effectiveness in correctly identifying septic cases. Furthermore, the RFC and LGBM models also demonstrated strong predictive reliability with PPV of 0.73 and 0.76, respectively, reinforcing their utility for sepsis detection.

Brier score and BSS evaluations reveal that the RFC model achieved the best performance with the lowest Brier Score (0.13) and highest BSS (15.79%), substantially outperforming the baseline LR model with a Brier score of 0.15. The GBC, LGBM, and XGBoost models also showed strong performance, each with a Brier score of 0.13 and BSS values of 12.54%, 10.38%, and 14.33%, respectively. In contrast, the AdaBoost classifier had the highest Brier score (0.22) and a negative BSS (-47.29%), indicating poorer performance. These findings highlight that RFC, GBC, LGBM, and XGBoost models provide both accurate and well-calibrated probabilistic estimates for sepsis, as detailed in Table 2.

In Figure 3, the receiver operating characteristic curves for the various machine learning models are assessed in the study, including the LR, linear discriminant analysis, RFC, GBC, AdaBoost classifier, XGBoost, and the LGBM models. These curves graphically represent the true-positive rate versus the false-positive rate for each model, elucidating their efficacy in differentiating sepsis from nonsepsis cases. The AUC values were also provided to gauge the predictive accuracy of each model.

The receiver operating characteristic curves depicted in Figure 3 provide a direct comparison of the model performances. These curves plot the true-positive rate against the false-positive rate and identify the ensemble models (GBC, RFC, and LGBM) that are particularly effective in differentiating between sepsis and nonsepsis cases, making them valuable tools for ICU diagnostics.

In summary, our findings suggest that the GBC, RFC, and LGBM models, with their high AUC values, balanced performance, and strong PPV scores, are robust tools for early sepsis prediction and can substantially enhance the clinical workflow by enabling timely and accurate sepsis interventions.

### Clinical Application of the Predictive Model

**Figure 4:**
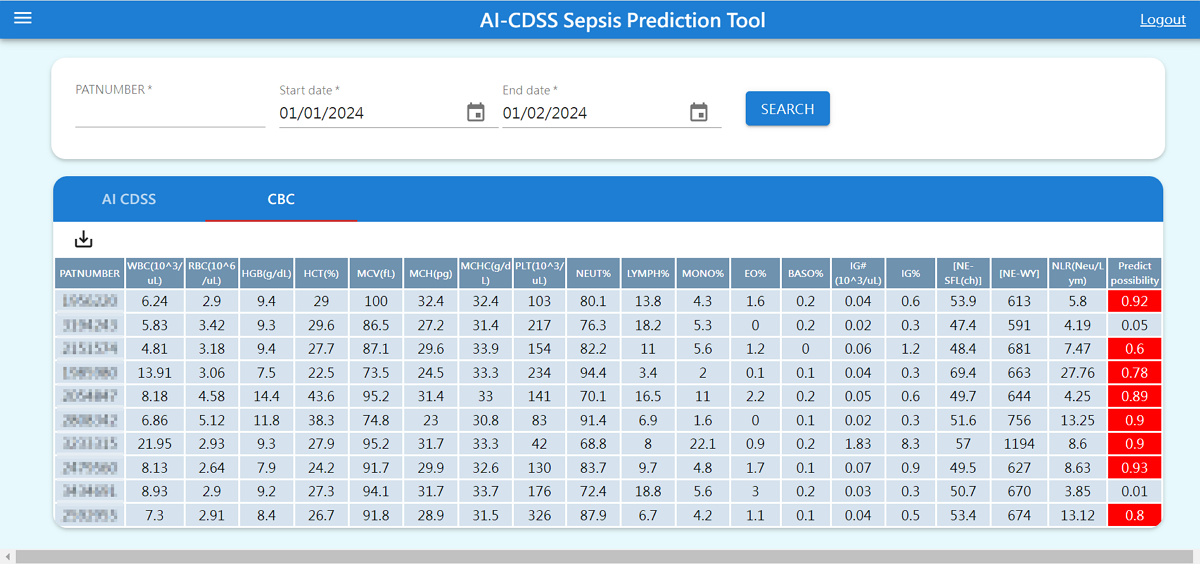
Interface demonstration for web-based sepsis risk assessment. A screenshot of the innovative web-based interface developed in this study. This interface was designed to allow health care professionals to load complete blood count data and swiftly assess sepsis risk. AI-CDSS: artificial intelligence–clinical decision support system.

Following a comprehensive evaluation, the LGBM model was selected as the backbone of our AI-CDSS owing to its superior performance on key metrics such as the AUC, sensitivity, and specificity. A key advancement in clinical decision-making is the development of a web-based interface for health care professionals. This tool allows for the quick input of CBC data to obtain an immediate assessment of the sepsis risk. A screenshot of this user-friendly functional interface is shown in Figure 4, demonstrating its applicability in real-time clinical settings. This interface marks a significant step forward in applying our machine learning model for swift and reliable sepsis risk evaluation, thereby optimizing the diagnostic process in ICU settings.

## Discussion

### Principal Findings

Our research contributes to advancements in rapid sepsis diagnostics through the development and validation of machine learning models that use CBC+DIFF data. The encouraging performance of the LGBM, GBC, and RFC models, as indicated by their AUC values during the testing phases, suggests a potential shift from traditional, more cumbersome diagnostic methods to a swift, data-driven approach using routine blood tests for the early detection of sepsis.

### Importance of Early Sepsis Detection and Advanced Diagnostic Technologies

Early detection of sepsis is crucial to prevent progression to severe sepsis and septic shock, improving patient outcomes and reducing mortality. The mortality rate for septic shock (38.5%) is higher than that for sepsis (32.2%) [4], highlighting the need for early intervention and treatment for sepsis. Early intervention can significantly reduce the length of hospitalization; lower health care costs; and most importantly, improve the survival rates of patients [1,9]. Enhanced diagnostic accuracy can be achieved using new parameters that provide information on the morphological and functional characteristics of leukocytes, reflecting their activation and functional activity in response to infections [17]. In our study, we use the Sysmex XN-9100 system, which uses fluorescence flow cytometry along with blood-cell membrane surfactant reagents and fluorescent dyes targeting nucleic acids and proteins. This advanced technology provides detailed insights into the morphological characteristics of neutrophils, lymphocytes, and monocytes, further enhancing our ability to detect and understand sepsis at the cellular level.

### Interpreting the Models’ Effectiveness

The strong efficacy of LGBM, GBC, and RFC models underscores the potential of regular CBC+DIFF data as a valuable yet underexplored tool for early sepsis detection. The ability of these machine learning models to distinguish between septic and nonseptic states is particularly critical in ICU settings, where prompt diagnosis is of paramount importance [18], as multiple studies endeavor to detect early sepsis using machine learning models [15]. To further understand the contribution of each feature, we used heat map visualizations and SHAP values. The heat map analysis highlighted significant features such as neutrophil percentage, lymphocyte percentage, and neutrophil-to-lymphocyte ratio from the CBC+DIFF data, and SHAP values offered an additional perspective on how each feature influenced the model’s predictions, enhancing clinical interpretability. In addition to the AUC, we evaluated the models using other metrics, including accuracy, sensitivity, specificity, PPV, NPV, and the *F*_1_-score. The LGBM model outperformed the RFC and GBC models in these metrics, showcasing its superior overall performance. Furthermore, we assessed the models using the Brier score and BSS. The Brier score for the RFC, GBC, and LGBM models was 0.13, indicating similar accuracy among these models. However, the BSS showed that the RFC model had a slightly higher value of 15.79%, compared to the GBC model’s 12.54% and the LGBM model’s 10.38%, reflecting better performance relative to the baseline LR model, despite the LGBM model outperforming the RFC model in other metrics. Therefore, the LGBM model was integrated into our AI-CDSS backbone core owing to its overall performance.

### Comparison to Previous Work

Our sepsis prediction model based on CBC+DIFF data provides a streamlined and efficient approach compared to existing models that require numerous physiological and clinical parameters from laboratory profiles, electronic health records, vital signs, and monitoring devices [13,14,19]. Unlike these complex models, our approach relies exclusively on readily available hematological data, making it highly accessible across various clinical settings, including non-ICU environments. Despite its simplicity, our model achieves an AUC of 0.90, surpassing the performance of more complex models that report AUCs ranging from 0.83 to 0.87, demonstrating that comprehensive predictive capability can be achieved using basic hematological parameters alone [13,14,19]. The model’s simplicity offers significant advantages in terms of speed, cost-efficiency, and ease of implementation, particularly in settings where extensive data collection is challenging. By enabling rapid risk assessment through routine blood work, our model provides relative real-time decision support that facilitates efficient patient management across diverse health care settings, from emergency departments to resource-constrained environments.

### Workflow Integration

Currently, our AI-CDSS web system is deployed on our hospital’s internal server after validation. Physicians can log in to the system with their account, input the patient’s medical record number, and select the relevant CBC+DIFF test values for analysis. The system analyzes the test data and provides an estimated probability of sepsis. This deployment allows for seamless integration into daily clinical workflows, enabling quick and accurate assessments that support timely and informed clinical decisions. Compared to traditional methods, even when physicians use the SOFA score to assess sepsis, they typically have to wait approximately 3 days to confirm a positive bacterial culture from blood or sputum samples. In contrast, our new AI-CDSS system can provide an earlier indication of infection within minutes, allowing physicians to confirm sepsis sooner and intervene earlier, thereby improving patient outcomes.

### Clinical Relevance and Implementation

Considering the complex interplay of sepsis-related mortality and underlying diseases [20], the development of more precise diagnostic tools, such as our machine learning models, is essential to improve the early detection and management of sepsis in ICU settings. Our research, centered on the LGBM, GBC, and RFC, aligns with the broader trend of integrating diverse machine learning models in health care [21], reflecting the growing significance of artificial intelligence in enhancing sepsis diagnostics across various hospital settings [15]. Incorporating these models into ICU practices aligns with the emerging trends in health care [7] and could show potential to transform sepsis diagnostics, offering a rapid and precise alternative to traditional diagnostic approaches with only a minimally invasive blood draw required. The models’ adoption is expected to enable early therapeutic intervention, thereby enhancing patient outcomes [15]. The creation of a user-friendly, web-based interface for health care professionals exemplifies the practicality of these models, facilitating real-time clinical decision-making and improving patient care.

In resource-limited countries, our study presents a potential innovation for sepsis management using machine learning for CBC+DIFF analysis, providing a rapid, effective alternative to traditional methods, essential where resource constraints cause life-threatening diagnostic delays [22]. Using routine blood tests, health care providers in these regions can rapidly identify sepsis risk, enabling quicker intervention and potentially reducing the heavy dependence on limited antibiotics [23]. This method could assist in areas with limited access to advanced diagnostic facilities. Our study highlights the potential global impact of artificial intelligence–enhanced blood analysis, offering a promising approach to enhance sepsis diagnosis and improving patient outcomes in under-resourced settings.

### Limitations

Although our findings are encouraging, there are some limitations to our study. The retrospective nature of data collection may introduce selection bias, and reliance on a singular data source (CBC+DIFF) could restrict the breadth of the models. In addition, this study was conducted at a single center, although supported by branches that refer to sepsis cases and focused exclusively on pneumonia-induced sepsis, which may limit the generalizability of the findings to other causes of sepsis. Although our results are promising, they are preliminary and require further confirmation through prospective studies in various clinical settings to verify their robustness and applicability.

### Future Research

We recommend conducting extensive validation studies to confirm each model’s generalizability and performing cost-benefit analyses to evaluate their impact on health care systems. As these models are integrated into clinical practice, it is essential to provide comprehensive training for health care professionals to ensure the optimal utilization of web-based tools. Expanding the model data inputs to encompass more patient-specific factors could further improve the accuracy of sepsis prediction.

### Conclusions

In summary, our study contributes to advancements in sepsis diagnostics by exploring the use of machine learning to interpret CBC+DIFF data for enhanced sepsis diagnosis. This innovative approach may represent a significant shift in critical care and has the potential to impact diagnostics in various health care settings, including outpatient and emergency departments. By aiming to enable faster and more precise detection of sepsis, these models could significantly contribute to global health care efforts against sepsis, potentially aiding in improvements in patient care and outcomes. This approach may offer benefits in resource-limited countries, where diagnosing sepsis with only CBC+DIFF data provides a crucial alternative in scenarios where traditional diagnostic means are lacking.

## Appendix

Multimedia Appendix 1 SHAP summary plot for feature importance in the sepsis prediction model. SHAP: Shapley additive explanations.

## Abbreviations

AI-CDSS: artificial intelligence–clinical decision support system
AUC: area under the curve
BSS: Brier skill score
CBC+DIFF: complete blood count with differential
GBC: gradient boosting classifier
ICU: intensive care unit
LGBM: light gradient boosting machine
LR: logistic regression
NPV: negative predictive value
PPV: positive predictive value
RFC: random forest classifier
SHAP: Shapley additive explanations
SOFA: Sequential Organ Failure Assessment
XGBoost: extreme gradient boosting

## Data Availability

The datasets analyzed during this study are available from the corresponding author on reasonable request.
