# Supplementary material for: AI-Driven Innovations for Early Sepsis Detection by Combining Predictive Accuracy With Blood Count Analysis in an Emergency Setting: Retrospective Study
Source: J Med Internet Res. 2025 Jan 24;27:e56155. doi: 10.2196/56155 (PMC11809270; doi:10.2196/56155)
Supplement: Multimedia Appendix 1 [file jmir_v27i1e56155_app1.docx]

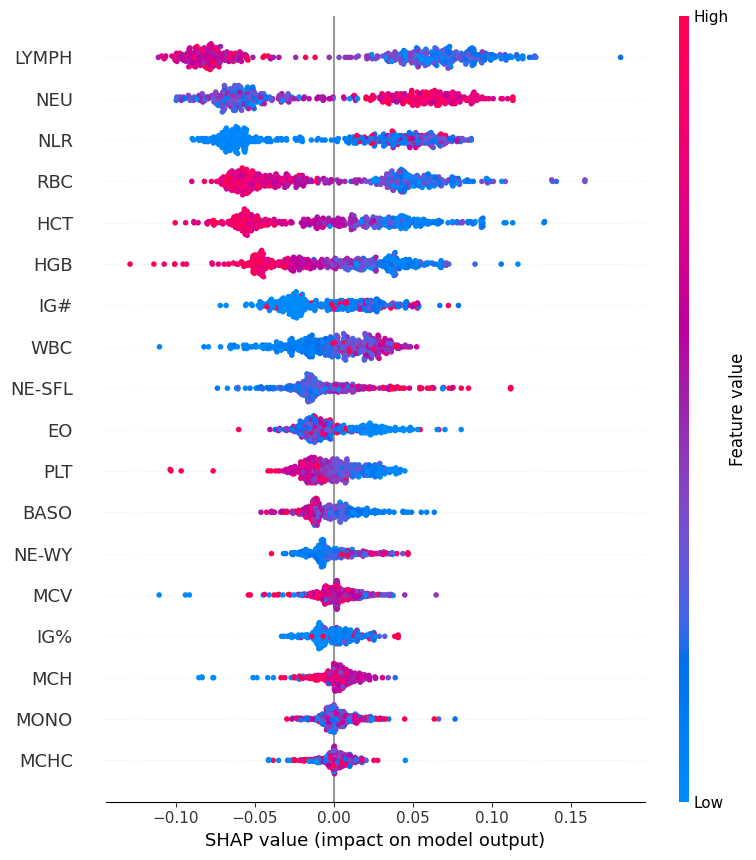


Supplementary Figure 1: SHAP Summary Plot for Feature Importance in Sepsis Prediction Model

This figure illustrates the SHapley Additive exPlanations (SHAP) summary plot, highlighting the impact of various blood count parameters on the predictive model for sepsis. Each dot represents a SHAP value for a particular feature of a patient, with colors indicating the feature value (red for high values and blue for low values). The features are arranged from top to bottom based on their importance, with LYMPH (Lymphocyte percentage), NEU (Neutrophil percentage), and NLR (Neutrophil to Lymphocyte Ratio) being the top contributors to the model’s output. The three least important features in the model are MCHC (Mean Corpuscular Hemoglobin Concentration), MONO (Monocyte percentage), and MCH (Mean Corpuscular Hemoglobin), indicating their minimal impact on the sepsis predictions, which aligned with the interpretation from Figure 2. This plot provides a visual representation of how different features contribute to the model's predictions, enhancing the interpretability and clinical relevance of the model used for early sepsis detection.
